# Leaping plastic thermoelectrics through multi-heterojunction design

**DOI:** 10.1093/nsr/nwae386

**Published:** 2024-10-29

**Authors:** Jing-Tao Lü

**Affiliations:** School of Physics, Huazhong University of Science and Technology, China

The interconversion of heat and electricity using thermoelectric effect has the potential to provide a sustainable energy supply from waste heat and to be used in precise temperature control [1]. The thermoelectric conversion efficiency is characterized by the dimensionless figure of merit $ZT = {{S}^2}\sigma T/\kappa $, with $S,\ \sigma ,\ \kappa ,T$ the Seebeck coefficient, the electrical conductivity, the thermal conductivity (including lattice ${{\kappa }_L}\ $and electron ${{\kappa }_e}$ contributions) and the absolute temperature, respectively. Desired thermoelectric applications require a *ZT* value larger than 1, which should be found in doped semiconductors.

Since the 1990s, two distinct research routes have emerged in pursuing state-of-the-art thermoelectrics. One avenue explores low dimensional structures, i.e. superlattices with multiple interfaces. Enhanced phonon scattering at these interfaces can suppress ${{\kappa }_L}$, while sharper energy dependence of the low-dimensional electron density of states promotes *S*. The other way focuses on advanced bulk materials, which show a phonon-glass/electron-crystal property, characterized by low phonon and high electrical transport coefficients. Very recently, the two approaches have been combined [2,3].

Organic thermoelectric materials offer the unique advantages of being low cost, light weight and having mechanical flexibility, enabling applications that are challenging for traditional thermoelectrics [1]. Organic semiconductors with high electrical conductivity are normally in the polycrystalline form with domains of different sizes and orientations, resulting in their low ${{\kappa }_L}$, close to phonon-glass. Thus, existing research has primarily focused on improving the electric power factor ($PF$) [4], i.e. through an optimized doping strategy. However, the figure of merit $( {ZT\ \sim 0.01 - 0.5} )$ remains much lower than that of commercial inorganic materials.

**Figure 1.**
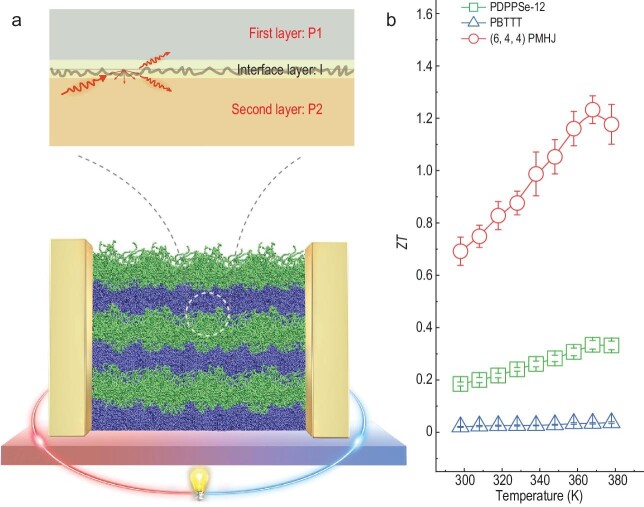
Designed polymeric multi-heterojunctions (PMHJs) with periodic dual-heterojunction features. The concept is similar to, but different from, inorganic superlattice structures. Each period of the PMHJ includes two distinct polymer layers, together with two interpenetrating interfaces. (a) Schematic illustration of the PMHJ film. (b) Several-fold increase of *ZT* for the PMHJ constructed from two types of polymers (PBTTT and PDPPSe-12), compared to the corresponding result from a single component. Reproduced with permission from ref. [5].

A recent breakthrough, published in *Nature* [5], has taken a different route. The team, led by Prof. Chong-an Di and Daoben Zhu from the Institute of Chemistry, Chinese Academy of Sciences in Beijing, reported a record-high *ZT* of 1.28 at 368 K in designed polymeric multi-heterojunctions (PMHJs) (details in caption of Fig. 1). The method developed by the authors allows precise manipulation of the polymer and interface thickness to sub-10 and sub-5 nm, respectively.

Crucial to the record-high *ZT* is the ∼60% decrease of the in-plane $\kappa $ compared to the individual polymer layer. While the reduction of out-of-plane $\kappa $ in superlattice structures is well understood, achieving a similar relative decrease in the in-plane direction is less expected. The authors attribute this surprising result to enhanced scattering of propagons, phonon-like heat carriers in polycrystalline structures, at the interpenetrating interfaces. In addition to the drastic reduction of in-plane $\kappa $, the mixing of the two types of polymers at the interface introduces more entropy to the electrons and results in higher *S*, compensating for the decreased$\ \sigma $ and resulting in an overall slightly increased *PF*.

The next step is to gain further understanding of the carrier transport mechanism in these quasi-2D polymer layers for further optimization of the *PF*. On the other hand, exploring different combinations of organic thermoelectric materials to build the PMHJ could potentially trigger further breakthroughs in thermoelectric performance, especially for *n*-type materials, which is a known challenge in organic thermoelectrics. Finally, optimization of the fabrication process is needed for practical applications using this design strategy.
